# Design and Field Methods of the ARISE Network COVID-19 Rapid Monitoring Survey

**DOI:** 10.4269/ajtmh.20-1618

**Published:** 2021-06-23

**Authors:** Elena C. Hemler, Michelle L. Korte, Bruno Lankoande, Ourohiré Millogo, Nega Assefa, Angela Chukwu, Firehiwot Workneh, Amani Tinkasimile, Isaac Lyatuu, Abdramane Soura, Dongqing Wang, Isabel Madzorera, Said Vuai, Till Bärnighausen, Mary Mwanyika Sando, Japhet Killewo, Ayoade Oduola, Ali Sie, Yemane Berhane, Wafaie W. Fawzi

**Affiliations:** 1 Department of Global Health and Population, Harvard T.H. Chan School of Public Health, Harvard University, Boston, Massachusetts;; 2 Institut Supérieur des Sciences de la Population, University of Ouagadougou, Ouagadougou, Burkina Faso;; 3 Nouna Health Research Center, Nouna, Burkina Faso;; 4 College of Health and Medical Sciences, Haramaya University, Harar, Ethiopia;; 5 Department of Statistics, University of Ibadan, Ibadan, Nigeria;; 6 Department of Epidemiology and Biostatistics, Addis Continental Institute of Public Health, Addis Ababa, Ethiopia;; 7 Africa Academy for Public Health, Dar es Salaam, Tanzania;; 8 College of Natural and Mathematical Sciences, University of Dodoma, Dodoma, Tanzania;; 9 Heidelberg Institute of Global Health, Heidelberg University, Heidelberg, Germany;; 10 Africa Health Research Institute, KwaZulu-Natal, South Africa;; 11 Department of Epidemiology and Biostatistics, Muhimbili University of Health and Allied Sciences, Dar es Salaam, Tanzania;; 12 University of Ibadan Research Foundation, University of Ibadan, Ibadan, Nigeria;; 13 Department of Nutrition, Harvard T.H. Chan School of Public Health, Harvard University, Boston, Massachusetts;; 14 Department of Epidemiology, Harvard T.H. Chan School of Public Health, Harvard University, Boston, Massachusetts

## Abstract

The coronavirus disease 2019 (COVID-19) pandemic has significant health and economic ramifications across sub-Saharan Africa (SSA). Data regarding its far-reaching impacts are severely lacking, thereby hindering the development of evidence-based strategies to mitigate its direct and indirect health consequences. To address this need, the Africa Research, Implementation Science, and Education (ARISE) Network established a mobile survey platform in SSA to generate longitudinal data regarding knowledge, attitudes, and practices (KAP) related to COVID-19 prevention and management and to evaluate the impact of COVID-19 on health and socioeconomic domains. We conducted a baseline survey of 900 healthcare workers, 1,795 adolescents 10 to 19 years of age, and 1,797 adults 20 years or older at six urban and rural sites in Burkina Faso, Ethiopia, and Nigeria. Households were selected using sampling frames of existing Health and Demographic Surveillance Systems or national surveys when possible. Healthcare providers in urban areas were sampled using lists from professional associations. Data were collected through computer-assisted telephone interviews from July to November 2020. Consenting participants responded to surveys assessing KAP and the impact of the pandemic on nutrition, food security, healthcare access and utilization, lifestyle, and mental health. We found that mobile telephone surveys can be a rapid and reliable strategy for data collection during emergencies, but challenges exist with response rates. Maintaining accurate databases of telephone numbers and conducting brief baseline in-person visits can improve response rates. The challenges and lessons learned from this effort can inform future survey efforts during COVID-19 and other emergencies, as well as remote data collection in SSA in general.

## INTRODUCTION

In November 2019, the first cases of the coronavirus disease 2019 (COVID-19) were reported in Wuhan, China.^1^ By February 2021, cases had surpassed 107 million, with more than 2.3 million deaths worldwide.^2^ The first case in Africa was recorded in Egypt on February 14, 2020, with the continent reaching 3.5 million cases and more than 88,000 deaths by February 2021.^3^ So far, much of sub-Saharan Africa (SSA) has been spared the catastrophic impact feared at the outset of the pandemic; however, COVID-19 continues to pose a substantial threat because of scarce public health resources, fragile health systems, infrastructural challenges, food insecurity, and a high prevalence of many other infectious diseases.^4^ In addition to the morbidity and mortality directly caused by COVID-19, the pandemic may have indirect adverse economic and health consequences in SSA, including those for health service access and utilization, physical health, mental health, food security, and education for children and adolescents. Understanding these far-reaching effects and current population-level knowledge and practices related to COVID-19 is fundamental to advancing prevention strategies and interventions to mitigate the consequences of this pandemic.

Evidence of the impact of the pandemic and the results of the prevention efforts imposed in SSA has been severely lacking. Many national and pan-African leaders have responded promptly to the COVID-19 threat, implementing preventive measures such as full and partial lockdowns, quarantines, travel bans, curfews, and school closures, which are supplemented by consistent and evidence-based messaging and rapid scaling of testing capacities.^5^ Although these efforts have restricted the spread of the virus, they have inadvertently disrupted livelihoods and suspended critical health and education services, posing serious immediate and long-term consequences that may exacerbate pre-existing inequities. Modeling efforts have demonstrated potentially devastating effects of the pandemic through disruption of routine health care services and decreased access to food, which could lead to a 9.8% to 44.7% increase in deaths of children younger than 5 years and an 8.3% to 38.6% increase in maternal deaths every month across 118 countries.^6^ In high-burden settings, HIV, tuberculosis, and malaria deaths over the next 5 years could increase by up to 10%, 20%, and 36%, respectively, because of reductions in timely diagnosis and treatment and the interruption of prevention campaigns.^4^ Additional modeling efforts have also suggested potential HIV-related mortality attributable to antiretroviral therapy disruption,^7^ child mortality attributable to the suspension of immunization campaigns,^8^ and increased malaria cases and deaths attributable to the disruption of key malaria control interventions.^9^

### Study rationale.

Although modeling studies are useful for predicting large-scale effects of the pandemic, few studies have examined the actual effects of COVID-19 on health service access and utilization or nutrition and food security in SSA. According to one qualitative study among slum communities in Kenya, Nigeria, Bangladesh, and Pakistan, impacts of COVID-19 included interrupted access to food, disruptions in health services offered, challenges in reaching healthcare facilities, increased costs of healthcare, reduced household incomes, fears of infection, and fears of stigmatization preventing healthcare-seeking.^10^ Anecdotal reports have suggested that there are nutrition and food security challenges in SSA because of COVID-19.^10,11^ However, few studies have quantified these impacts. One online survey performed in Kenya and Uganda found worsening food insecurity and diet quality, and that more than two-thirds of respondents reported income shocks caused by the pandemic.^12^ Another study examined the potential impacts of movement restrictions created to limit the spread of COVID-19 and predicted that these may disrupt farming activities, resulting in potential negative impacts on agricultural production and food security.^11^

The COVID-19 pandemic has also resulted in the largest disruption of education systems to date, including the loss of school meals for approximately 370 million children worldwide.^13^ These impacts may be particularly detrimental to adolescents who are in a critical period of development and may experience significant threats to their diets, lifestyles, and mental health; and the damage from these impacts may be difficult to reverse.^14^ Although the virus may be less severe among adolescents than among older populations,^15^ adolescents may have a key role in transmitting the virus to more vulnerable individuals.^16^ Therefore, it is necessary to fill the knowledge gap regarding knowledge, attitudes, and practices (KAP) related to COVID-19 among adolescents. It is also critical to understand impacts of the pandemic and preventive measures imposed on adolescent health and wellbeing, because health in adolescence sets the trajectory for health throughout the life course.

Investigating KAP related to COVID-19 can help to identify gaps and inform future education and behavior change interventions targeting the general population as well as healthcare providers. Of the few studies that have measured COVID-19 KAP in SSA, many had limitations in their sampling procedures (e.g., through facility-based^17,18^ or Internet-based recruitment^19^) and population segments surveyed (e.g., with unrepresentative or small sample sizes^20^), and very few multi-site studies have been conducted to examine potential differences across countries. Some studies among the general population have found low knowledge of COVID-19 in countries such as the Democratic Republic of the Congo.^21^ However, others have identified low compliance with recommended health measures and preventive practices despite knowledge of COVID-19 in countries such as Egypt, Nigeria, and Sudan.^20,22,23^ A study performed in Sierra Leone found a strong association between knowledge and practices, but that deficits in each leave many people at risk.^24^ Among healthcare providers, most current data regarding KAP as well as experiences dealing with COVID-19 in facilities and communities are limited to certain specialties in specific locations.^25–28^

To fill these evidence gaps for health service access and utilization, food insecurity and nutrition, adolescent experiences and education, and populations’ KAP related to COVID-19 prevention and management, the Africa Research, Implementation Science, and Education (ARISE) Network established a novel mobile survey platform to generate data from three key population groups: adults and adolescents from the general population and healthcare providers. This mobile survey platform builds on lessons learned from mobile telephone surveys during past crises and their ability to produce real-time data to monitor impacts and facilitate timely and effective interventions. During the 2014 Ebola outbreak in West Africa, mobile telephone-based surveys contributed to an enhanced understanding of the impact of the outbreak on all-cause morbidity and mortality as well as health-seeking behavior.^29–31^ Similarly, this survey aims to quantify the impacts of the COVID-19 pandemic as well as populations’ knowledge of COVID-19 and engagement in preventive measures.

This project leveraged the partnerships and infrastructure of the ARISE Network which includes 21 member institutions from nine sub-Saharan African countries. The baseline survey included six sites across Burkina Faso, Ethiopia, and Nigeria, with an intent to longitudinally follow-up with participants over time and expand future additional survey rounds across other ARISE Network sites. African leaders have expressed the need for more disaggregated data regarding the health and economic impacts of this crisis to inform focused and prioritized efforts to maximize impact amid severe constraints on time and resources.^32^ Findings from this survey will provide decision-makers with data and tools to facilitate more effective and targeted interventions and strengthen their responses to the COVID-19 crisis.

## MATERIALS AND METHODS

### Setting and study population.

The ARISE COVID-19 Survey was developed in partnership with the Africa Academy for Public Health (Tanzania), the Center de Recherche en Santé de Nouna (Burkina Faso), University of Ouagadougou (Burkina Faso), Haramaya University (Ethiopia), Addis Continental Institute of Public Health (Ethiopia), Muhimbili University of Health and Allied Sciences (Tanzania), University of Dodoma (Tanzania), University of Ibadan Research Foundation (Nigeria), Heidelberg Institute of Global Health (Germany), and Harvard T.H. Chan School of Public Health (United States). Sites were selected based on the existing data collection infrastructure, willingness of site leaders to take on a new survey, and research capacity, including collecting, managing, and storing high-quality survey data, conducting analyses, and performing dissemination of findings.

**Figure 1.**
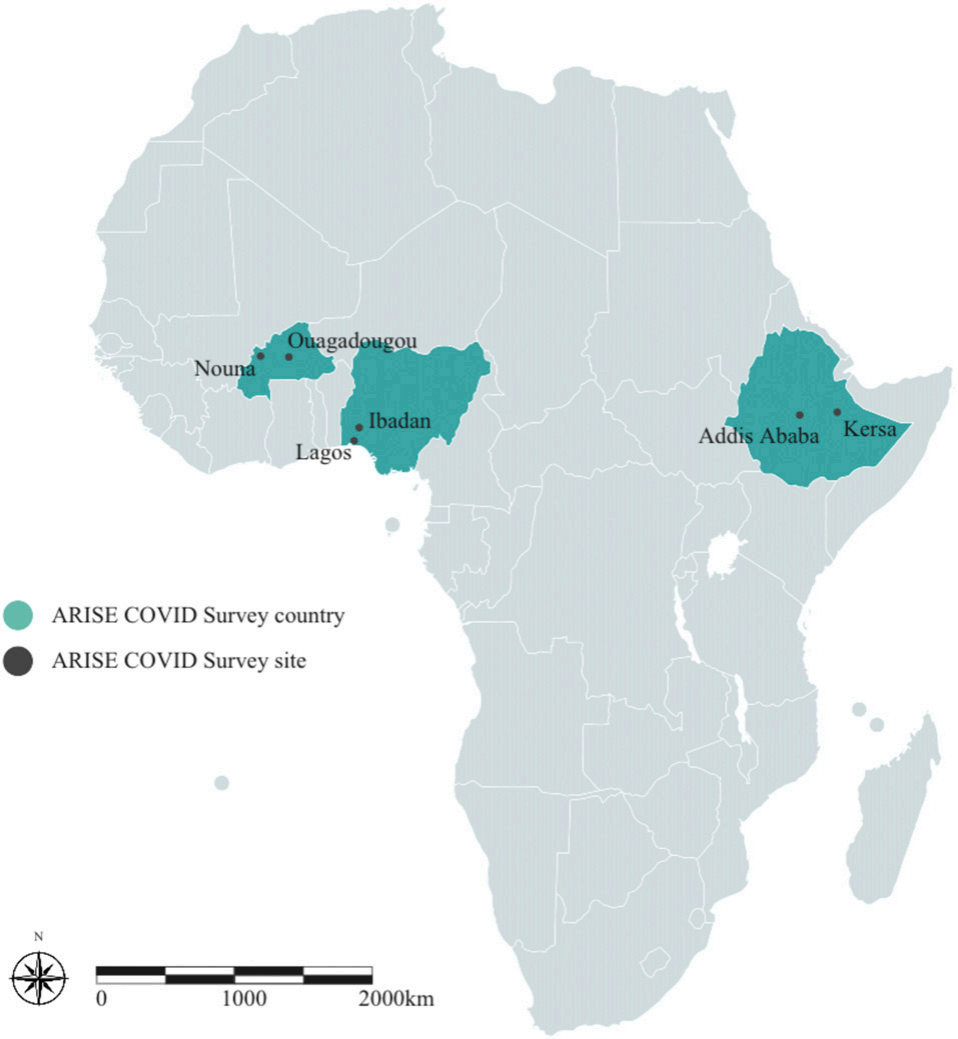
Map of ARISE Network COVID-19 study communities. This figure appears in color at www.ajtmh.org.

Investigators at each site selected the specific study communities, including one urban and one rural area, within each country to understand the potentially different impacts of COVID-19 on different settings. The urban study communities were in Ouagadougou in Burkina Faso, Addis Ababa in Ethiopia, and Lagos in Nigeria. The rural communities were in Nouna in Burkina Faso, Kersa in Ethiopia, and Ibadan in Nigeria (Figure 1). Sites were categorized as urban or rural based on how the communities are categorized administratively according to the governments of each country. The communities included in the study represent a wide variety of contexts, thus offering valuable opportunities for comparative research. In addition to the purposeful selection of communities based on heterogeneity and the opportunity for comparisons, we also chose the communities based on our ability to rapidly deploy a novel survey. The two main factors in our assessment of whether we could rapidly deploy the survey were an existing research infrastructure for survey science that was available to us, including data collectors and health researchers familiar with the community, tablets for electronic data collection, and systems to collect and store electronic data, and previous experience working in the community. In each country, healthcare workers, adolescents, and adults from the general population were recruited for the study. Demographic characteristics of adults and adolescents enrolled in the household survey in each community are shown in Table 1. Demographic characteristics of the healthcare providers are shown in Table 2.

**Table 1:** Demographic characteristics of 1797 adult participants and 1795 adolescent participants enrolled in each community in the ARISE Network COVID-19 Survey

Adults						
	Burkina Faso, Nouna (rural)	Burkina Faso, Ouagadougou (urban)	Ethiopia, Kersa (rural)	Ethiopia, Addis Ababa (urban)	Nigeria, Ibadan (rural)	Nigeria, Lagos (urban)
	*N* = 297	*N* = 300	*N* = 297	*N* = 288	*N* = 304	*N* = 311
Age, years, mean ± SD	48.4 ± 13.1	47.3 ± 9.9	36.7 ± 7.6	38.8 ± 12.6	41.4 ± 12.2	40.8 ±12.9
Sex, N (%)						
Male	262 (88.2)	204 (68.0)	231 (77.8)	102 (35.4)	148 (48.7)	192 (61.7)
Head of household, N (%)						
Yes	258 (86.9)	260 (86.7)	253 (85.2)	227 (78.8)	154 (50.7)	188 (60.5)
Role in household, N (%)						
Mother/wife	27 (9.1)	95 (31.7)	56 (18.9)	150 (52.1)	138 (46.6)	88 (30.1)
Father/husband	252 (84.8)	200 (66.7)	241 (81.1)	88 (30.6)	118 (39.9)	162 (55.5)
Other	18 (6.1)	5 (1.7)	0 (0.0)	50 (17.4)	40 (13.5)	42 (14.4)
Household size, mean ± SD	9.9 ± 5.0	7.3 ± 3.0	7.0 ± 2.2	4.2 ± 1.7	5.3 ± 2.5	4.9 ± 2.2
Children younger than 5 years in the household, mean ± SD	1.4 ± 1.5	0.9 ± 1.0	1.3 ± 1.0	0.5 ± 0.7	0.6 ± 0.9	0.5 ± 0.9
Highest level of education, N (%)						
None	183 (61.6)	174 (58.0)	106 (35.7)	29 (10.1)	6 (2.0)	2 (0.7)
Some primary	46 (15.5)	39 (13.0)	115 (38.7)	73 (25.3)	5 (1.7)	0 (0.0)
Completed primary	36 (12.1)	31 (10.3)	40 (13.5)	27 (9.4)	45 (15.0)	15 (4.9)
Some secondary	23 (7.7)	50 (16.7)	23 (7.7)	45 (15.6)	39 (13.0)	5 (1.6)
Completed secondary	3 (1.0)	1 (0.3)	11 (3.7)	43 (14.9)	82 (27.2)	49 (16.1)
Tertiary or higher	6 (2.0)	5 (1.7)	2 (0.7)	70 (24.3)	121 (40.2)	230 (75.7)
Adolescents 10 to 19 years of age						
	*N* = 297	*N* = 300	*N* = 294	*N* = 296	*N* = 365	*N* = 243
Age, years, mean ± SD	15.7 ± 2.5	15.1 ± 2.7	14.4 ± 3.1	16.1 ± 2.3	14.8 ± 2.3	16.1 ± 2.7
Sex, N (%)						
Male	157 (52.9)	139 (46.3)	200 (68.0)	102 (34.5)	148 (40.5)	114 (46.9)
Highest level of education, N (%)						
None	60 (20.2)	13 (4.3)	6 (2.0)	3 (1.0)	0 (0.0)	2 (0.8)
Some primary	81 (27.3)	69 (23.0)	212 (72.1)	101 (34.1)	11 (3.0)	3 (1.2)
Completed primary	40 (13.5)	49 (16.3)	19 (6.5)	41 (13.9)	102 (27.9)	42 (17.3)
Some secondary	107 (36.0)	157 (52.3)	47 (16.0)	85 (28.7)	221 (60.5)	118 (48.6)
Completed secondary	7 (2.4)	12 (4.0)	5 (1.7)	41 (13.9)	26 (7.1)	59 (24.3)
Tertiary or higher	2 (0.7)	0 (0.0)	5 (1.7)	25 (8.4)	5 (1.4)	19 (7.8)

**Table 2:** Demographic characteristics of 900 healthcare providers enrolled across urban communities in the ARISE Network COVID-19 survey

Healthcare providers			
	Burkina Faso (Ouagadougou)	Ethiopia (Addis Ababa)	Nigeria (Lagos, Ibadan)
	*N* = 300	*N* = 300	*N* = 300
Age, years, mean ± SD	39.7 ± 9.9	34.4 ± 10.5	45.3 ± 9.2
Sex, N (%)			
Male	157 (52.3)	141 (47.0)	74 (24.7)
Healthcare field (N, %)			
Medicine	83 (27.7)	114 (38.0)	78 (26.0)
Nursing	217 (72.3)	183 (61.0)	220 (73.3)
Other (specify)	0 (0.0)	3 (1.0)	2 (0.7)
Type of facility, N (%)			
Government hospital/clinic	161 (53.7)	211 (70.3)	253 (84.9)
Private hospital/clinic	71 (23.7)	63 (21.0)	44 (14.8)
Health outpost	25 (8.3)	2 (0.7)	1 (0.3)
Other	43 (14.3)	24 (8.0)	0 (0.0)

### Communities and sampling procedures.

#### Household survey.

To survey adults and adolescents, sites used different sampling frames, depending on the platforms available, including the following: existing Health and Demographic Surveillance Systems (HDSS) in Burkina Faso and rural Ethiopia (Kersa); Nigeria Living Standards Survey (NLSS) 2018–2019 and telephone service providers in Nigeria; and a new household survey we established in urban Ethiopia (Addis Ababa). Eligible potential participants in all communities were randomly selected from household and household member sampling frames derived from the HDSS or other recent census records (except for Addis Ababa, where a new census was conducted).

#### Healthcare workers.

The sampling frames for healthcare providers were constructed by obtaining lists from major medical professional associations in each country and narrowing the lists to focus on providers in urban areas (in Ouagadougou, Addis Ababa, Lagos, and Ibadan). We chose to focus on providers only in urban areas because of practical considerations of higher numbers of providers in urban areas and increased availability of contact information for these providers compared with those in rural areas. Healthcare workers were eligible to be included in the study if they were currently working in a healthcare setting. Clinicians from both public and private health facilities were recruited; there were no restrictions regarding medical specialties or whether they were providing COVID-19-related services. Dentists, pharmacists, and other allied health professionals (such as therapists and dietitians) were excluded.

### Field methods.

The target sample sizes per site were 300 healthcare providers from urban areas of each of the three countries (for a total of 900 providers), and 300 adults and 300 adolescents from each of the six sites (1,800 adults and 1,800 adolescents in total). From each sampling frame (urban household, rural household, and healthcare providers), additional telephone numbers beyond the target sample size were selected to allow for nonresponse or refusal. We aimed to select 500 healthcare providers from each country to allow for a 60% nonresponse rate, but higher rates of nonresponse in some countries led us to select additional healthcare providers from the sampling frames at some sites to reach the target sample size. Based on HDSS records and previous survey experience at these sites, for the household survey, we assumed that 20% of the households had adolescents and that 60% of the selected households would respond to the survey. This led us to aim to randomly select 2,500 households from each urban site and rural site to reach the minimum target of 300 adolescents and 300 adults from each site. Some sites were able to use prior census records to select households with known adolescents. From each household, we selected one adult 20 years or older and one adolescent 10 to 19 years of age (this age group was defined based on the World Health Organization definition of adolescence)^33^ if there was at least one adolescent regularly residing in the household.

Data collectors called each household to obtain consent to survey the household head or another person in the household and to obtain parental/guardian permission to speak to the adolescent if one was present in the household. After the adult completed the survey, data collectors obtained assent from the adolescent and completed the adolescent interview or arranged to call the adolescent at a later time. When two or more adults or adolescents were listed for one household, one was selected randomly for interview by selecting the participant with the nearest birthday. Before obtaining informed consent, data collectors screened participants to ensure they were eligible and in the correct age range. After obtaining consent, they directly asked each participant to provide their age in years. Specific procedures for each site are described.

#### Nouna, Burkina Faso.

Nouna is a semi-urban town in the northwest of Burkina Faso that serves as the administrative center of the Kossi province. Two previous ARISE surveys have been conducted in Nouna.^34–37^ Established in 1992, the Nouna HDSS includes the town of Nouna and surrounding villages.^38^ Composed primarily of rural areas, the HDSS includes approximately 124,960 inhabitants and is distributed over 1,775 km^2^. Trained field staff members visit all households within the HDSS boundaries every 4 months to record births, deaths, in-migration, and out-migration. Telephone numbers were collected from each household in 2019. For this study, 2,500 households were randomly sampled from the overall list of 15,014 households enrolled in the HDSS. Households were called and surveyed until the target sample size for adults was reached. After reaching the target sample size for adults, additional households from the sampling frame were called to determine if there was an adolescent present, and only adolescents from those households were surveyed until the sample size for adolescents was also reached. Of the sampled households called, 75% of telephone numbers were not functioning or were incorrect. A total of 2,427 households were called to reach the final sample of 297 adults and 297 adolescents. Data were collected during the rainy season in August to September 2020.

#### Ouagadougou, Burkina Faso.

Ouagadougou is the largest city in Burkina Faso and the country’s capital. The Ouagadougou HDSS was established in 2008, and is located in five neighborhoods at the northern periphery of the city.^39^ Data regarding vital events (births, deaths, unions, and migrations) are collected during household visits every 10 months. The HDSS neighborhoods include formal areas (approximately 40,000 residents) and informal neighborhoods devoid of formal zoning plans (approximately 40,000 residents) to target the most vulnerable populations in the city. The HDSS includes 20,274 households, of which 10,627 included adolescents 10 to 19 years of age. For this survey, study staff randomly selected 2,500 households on the list of households with adolescents. Every household included in the survey had data from one adult and one adolescent. Approximately 1,200 households were called to reach the final sample size of 300 adolescents and 300 adults.

For the healthcare provider survey, names and telephone numbers were obtained from professional physician and nursing associations. The lists included 1,091 nurses and 3,577 physicians. To account for nonresponse, 242 nurses and 330 physicians were sampled from each list. These sampling numbers were determined based on experiences during a pilot survey and difficulties obtaining physician responses. To reach the final sample size of 300 healthcare providers, 225 nurses and all 330 physicians were called. The household survey and healthcare providers survey were completed during the rainy season in August to September 2020.

#### Kersa, Ethiopia.

Kersa is a rural district of Ethiopia located in the east Hararghe zone of the Oromia region. The Kersa HDSS is managed by Haramaya University and was established in 2007; it has served as a site for previous ARISE studies.^34,40^ It is an open cohort of all individuals permanently living in 24 of the 38 sub-districts of Kersa.^41^ Approximately 131,000 individuals are currently undergoing surveillance. The Kersa HDSS records births, deaths, in-migration, and out-migration every 6 months; it also records other information, including changes in marital status, pregnancy outcomes, health status, and economic status at longer intervals. Household telephone numbers were not available in the Kersa HDSS; therefore, household visits were conducted in the 24 sub-districts of the study site to identify 500 voluntary adults and 500 voluntary adolescents to participate in the telephone survey. To sample households for this survey from the overall list of all 27,882 HDSS households, accessible villages were first identified (because some villages were not accessible during the rainy season), and households in these villages with adults and adolescents were listed. From this list, a total of 1,700 households were randomly selected to reach 500 adults and 500 adolescents. Selected households were approached to determine if they had a working telephone and to obtain consent from the adults and adolescents. Each household visit was approximately 5 to 7 minutes in duration, and study staff members were equipped with personal protective equipment. During each household visit, study staff members provided a brief explanation of the content of the survey and objectives. Informed consent was secured from the adult participant and adolescent. Up to three telephone numbers for each household were recorded during the visit. If the adolescent had a separate telephone number, then that was also recorded. Then, each household was called to complete the interview. To reach the target sample size of 297 adult participants and 294 adolescent participants, 388 households were called. Data were collected during the rainy season from July to August 2020.

#### Addis Ababa, Ethiopia.

Addis Ababa is the largest city in Ethiopia and the country’s capital. There are no existing HDSSs in Addis Ababa; therefore, site leaders established a new household sampling frame for this study. Stratified multi-stage sampling was used to select three of the total of 10 sub-cities in Addis Ababa. Based on the daily COVID-19 caseload, three sub-cities were selected to represent low, medium, and high caseload sub-cities (according to the caseload reported on June 25, 2020, by the Ministry of Health and the Ethiopian Public Health Institute). Two woredas (the lowest administrative level in Addis Ababa) were then selected randomly from each sub-city and a census was conducted by visiting every other household in each woreda to obtain a list of 2,500 households. Each of the 2,500 households was visited to obtain telephone numbers and list family members. Each household visit was approximately 15 minutes in duration, and study staff members were equipped with personal protective equipment. During each household visit, study staff members provided a brief explanation of the content of the survey and objectives. Informed consent was secured from the adult participant, and parental permission and assent were obtained for the adolescent (if one was present in the household). Then, each household was called to complete the interview. A total of 919 households were called to reach the final sample of 288 adults and 296 adolescents.

For the healthcare provider survey, names and telephone numbers of healthcare providers were obtained from Ethiopian medical and nursing associations. The survey team sampled 300 physicians and 200 nurses from these lists. Some associations were not able to share telephone numbers for confidentiality reasons; therefore, to recruit additional participants from these lists, information about the survey was distributed to the associations’ members with an Internet link allowing them to opt into the telephone calls. All 500 physicians and nurses sampled from the Ethiopian medical and nursing associations and those who responded via the Internet link were called. However, using these methods, we were only able to complete 130 interviews with healthcare providers. Therefore, to obtain additional providers to reach the final sample of 300, we used snowball sampling by distributing the opt-in link to healthcare providers associated with Addis Continental Institute of Public Health and their networks. The household survey and healthcare providers survey were both completed from August to November 2020.

#### Ibadan, Nigeria.

Ibadan is the capital of the Oyo State in Nigeria and is composed of both urban and rural areas. Previous ARISE studies have been conducted in Ibadan.^34^ Telephone numbers of households in rural areas were obtained from the Nigeria Living Standards Survey (NLSS), which was a national survey of 22,110 households conducted from September 2018 to October 2019, by the National Bureau of Statistics of Nigeria to measure living conditions of the population of Nigeria.^42^ For this study, 1,200 households were randomly sampled from the NLSS list of 2,300 rural households in Ibadan. A short message service (SMS) message was sent to all sampled telephone numbers to alert them to expect a telephone call. All 1,200 households were called to reach the final sample size of 304 adults. A large proportion of households called did not have adolescents, or the adolescents were at school or not present at home to be interviewed; therefore, to reach the final sample size of 365 adolescents, we were required to supplement the NLSS lists with lists of telephone numbers from a previous adolescent survey that included 2,000 adolescents who were randomly selected from more than 20 rural and urban secondary schools in Ibadan.

#### Lagos, Nigeria.

Lagos is the largest city in Nigeria, and it is one of the largest cities in SSA. Data from HDSS and previous surveys were not available in Lagos; therefore, for this survey, we obtained 5,000 telephone numbers for households located in urban areas of Lagos from telephone service providers. From this list, 2,600 households were sampled. An SMS message was sent to all sampled telephone numbers to inform them of the study and alert them to expect a telephone call. All 2,600 households were called to reach the final sample size of 311 adults. As was the case in Ibadan, a large proportion of households called did not have adolescents, or the adolescents were not available to be interviewed. Therefore, we supplemented the lists from the telephone service providers with telephone numbers obtained from the STWG-MNCWH Adolescent Health and Education Survey conducted by the University of Ibadan Research Foundation in February 2020, to assess the state of adolescent health and education in the southwest geopolitical zone of Nigeria. This survey recruited 313 adolescents 10 to 19 years of age who were randomly sampled from secondary schools in Lagos and Ibadan in Nigeria. We called all 313 adolescents enrolled in this study to reach the final sample size of 243 adolescents enrolled in our survey.

For the healthcare provider survey, 5,500 names and telephone numbers of healthcare providers in urban areas of Lagos and Ibadan were obtained from physician and nursing associations. To account for high rates of nonresponse among healthcare providers in Nigeria, 1,000 physicians and 800 nurses were sampled from these lists. SMS messages were sent to all sampled telephone numbers to alert them to expect a telephone call. To reach the final sample size of 300 healthcare providers, 1,000 physicians and 600 nurses were called. Data for the healthcare provider survey and household survey in Lagos and Ibadan were collected during the rainy season from October to November 2020.

### Ethics and Informed Consent.

Before participating in the survey, all participants were first informed of the purpose and nature of the study. Verbal informed consent was obtained from all participants 18 years or older. Verbal parental permission and adolescent assent were obtained for adolescents younger than 18 years. At the end of the interview, all participants were connected to country-specific resources providing information regarding COVID-19. Data collectors were also trained to provide specific information regarding outreach services to those who reported experiencing mental health problems or physical abuse. Data collectors were trained to maintain confidentiality of all participant data, and the consent script described precautions to ensure confidentiality, including storing all data in a secure electronic database.

This survey was approved by all necessary ethical review boards in each country, including the Harvard T.H. Chan School of Public Health Institutional Review Board, Nouna Health Research Center Ethical Committee and National Ethics Committee in Burkina Faso, the Institutional Ethical Review Board of Addis Continental Institute of Public Health in Ethiopia, and the University of Ibadan Research Ethics Committee and National Health Research Ethics Committee in Nigeria. We originally planned to collect data at two additional sites in Tanzania; however, ethical approval from the National Institute for Medical Research was not received.

### Questionnaire.

Computer-assisted telephone interviews (CATI) were conducted by trained interviewers with all consenting participants using standardized questionnaires appropriate for each country and setting. The questionnaires were translated into local languages by experienced translators and checked by investigators at each site. Separate questionnaires for healthcare providers, adults, and adolescents were developed by working groups comprising experts from the ARISE Network and Harvard T.H. Chan School of Public Health across the domains covered by the research, including nutrition, mental health, health systems, substance use, and other areas. These experts also provided important local expertise regarding context and culture in each study community to ensure that response categories captured common attitudes across each setting. The final instrument was agreed upon by the multi-disciplinary team of research scientists involved in this study.

Table 3 displays the domains, topics, and specific questionnaire items included in each of the three instruments. The full instruments are also included as Supplemental Materials. The healthcare provider survey assessed practices related to COVID-19 prevention approaches at health facilities, knowledge and attitudes toward the virus, access to personal protective equipment and experiences in the workplace, stress and mental health, and impacts on health services. The survey for adults and adolescents assessed KAP related to COVID-19 prevention and management such as physical distancing, handwashing, and coughing practices. The survey also examined the impact of the outbreak on other health and nutrition domains such as food security and hunger, mental health, and access to vital medications, curative services, and preventive services such as antenatal care and immunization. We also assessed perceptions of the pandemic and the impact of school closures on adolescent health and well-being. To assess dietary diversity and quality and changes caused by the pandemic, we included an assessment of the consumption of food groups to allow for the computation of dietary diversity scores and a food-based dietary score (the Prime Diet Quality Score) differentiating healthy foods from unhealthy foods based on associations with chronic diseases.^43^ To assess food security, questions were adapted from the Household Food Insecurity Access Scale (HFIAS), which has been validated for measuring food access in developing country contexts.^44^ To assess mental health, we included the Patient Health Questionnaire 4 (PHQ-4), which is a validated instrument used to assess depression and anxiety.^45^ Select questions to assess KAP and perceptions related to the COVID-19 pandemic were also adapted from previous surveys.^46–48^

**Table 3:** Modules included in the ARISE Network COVID-19 survey adult, adolescent, and healthcare provider questionnaires

Adult general population survey		
Domain	Subtopic	Items
Socio-demographics	Demographics	Age
		Sex
		Role in household
		Household composition
		Educational attainment
		Occupation
COVID-19 knowledge, attitudes, practices	Knowledge	Belief in COVID-19
		Identification of symptoms
		Identification of transmission methods
		Identification of prevention measures
		Knowledge of someone with COVID-19
		Sources of information about the pandemic
	Attitudes	Maximum time could comply with a lockdown
	Practices	Factors preventing self-quarantine
		Preparation and response measures taken
Mental health and well-being	Health habits	Drinking habits during the past 2 weeks compared with usual habits
		Sleeping habits during the past 2 weeks compared with usual habits
	Anxiety and depression	Patient Health Questionnaire 4 (PHQ-4)
Healthcare utilization	Access to services	Childhood immunization
		Vitamin A supplementation for children
		Child health and nutrition prevention services
		Child malnutrition management
		Antenatal care
		Iron and folic acid for pregnant women
		HIV treatment
		Sexual and reproductive health
		Surgeries
		Reasons for disruptions in access
Nutrition and food security	Child feeding	Complementary feeding habits and impacts of the pandemic
		Breastfeeding practices and impacts of the pandemic
		Access to meals at school for children
	Impacts of COVID-19 on food pricing	Prices of staple foods
		Prices of pulses
		Prices of fruits
		Prices of vegetables
		Prices of animal source foods
	Food security over the past month	Skipping meals, going without eating for 1 day, and worrying about food running out
	Assistance received	Food aid
		Cash transfers
		School meals
	Farming	Impacts of the pandemic on crop production
	Diet quality	Prime Diet Quality Score/dietary diversity assessing number of days each food group was consumed over the past 7 days and number of days per week each food group was consumed during the time period before the coronavirus
Water, Sanitation and Hygiene (WASH)	Access	Access to safe and clean water for preparing food
		Access to soap
		Access to water for handwashing
Adolescent survey		
Descriptive characteristics	Demographics	Age
		Sex
		Education
		Occupation
COVID-19 knowledge, attitudes and practices	Knowledge	Belief in COVID-19
		Identification of symptoms
		Identification of transmission methods
		Identification of prevention measures
		Sources of information about the pandemic
	Attitudes	Concerns about coronavirus spread
		Perceived level of risk of coronavirus exposure
		Maximum time could comply with lockdown
	Practices	Preparation and response measures taken
Lifestyle	Impacts of COVID-19 on daily activities	Impacts on going to school, income, amount of time spent at home, responsibilities at home, and weight
	Physical activity	Level of physical activity before the pandemic and during the past 1 week
Education	COVID-19 impacts on education	School enrollment
		School closures because of the pandemic
		Methods of receiving education
		Coronavirus impacts on ability to learn
		Ability to catch up on education after the pandemic
		Meal provision at school
Communication	COVID-19 impacts on communication	Frequency of communication
		Main modes of communication
	Media consumption	Changes in media consumption
		Hours spent consuming media over the past 7 days
Nutrition	Diet assessment	Number of days per week consuming staples, pulses, fruits, vegetables, and animal source foods before coronavirus and during the past 7 days
Mental health and well-being	Sleeping habits	Sleeping habits during the past 7 days compared with usual habits
	Anxiety and depression	Patient Health Questionnaire 4 (PHQ-4)
Healthcare provider survey		
Descriptive characteristics	Demographics	Age
		Sex
		Occupation
		Type of healthcare facility
COVID-19 knowledge, attitudes, practices	Knowledge	Identification of symptoms
		Identification of transmission methods
		Identification of prevention measures
	Attitudes	Concern about coronavirus spread
		Perceived level of risk of exposure to coronavirus
		Perceived severity of the virus
	Practices	Experience caring for COVID-19 patients
Mental health and well-being	Health habits	Drinking habits during the past 2 weeks compared with usual habits
		Sleeping habits during the past 2 weeks compared with usual habits
	Anxiety and depression	Patient Health Questionnaire 4 (PHQ-4)
COVID-19 in the workplace	Access to prevention measures	Wearing masks
		Personal protective equipment
		Handwashing
		Distancing
		Sanitizers
		Regular cleaning and decontamination
	Training offered	Natural course of COVID-19
		Management and treatment
	Workplace policies	Presence of formal policies related to COVID-19
		Protocol for suspected cases
		Guidelines on delivering other health services to patients with COVID-19
Healthcare impacts	Impact on healthcare services	Childhood immunizations
		Vitamin A supplementation for children
		Child health and nutrition preventative services
		Management of child malnutrition
		Antenatal care
		Iron and folic acid for pregnant women
		HIV treatment services
		Sexual and reproductive health
		Surgeries
	Impact on prescription patterns	Antibiotics
		Antimalarials
		Multivitamins and other nutritional supplements
Stigma	Experiences as a healthcare professional	Social avoidance or rejection
		Denial of healthcare, education, housing, etc.
		Physical violence
		Congratulations or acknowledgment

The same questionnaires were implemented across sites with slight modifications when necessary to capture site-specific context. Many questions were close-ended with predefined response categories, but certain questions also allowed for specifying responses not captured by the response categories. After obtaining ethical clearance, a pilot study was performed in each country before the actual data collection to ensure adequate comprehension and usability of each instrument, allowing for feedback and minor adaptations before implementation.

### Data collection and management.

Trained research assistants (male and female) with prior data collection experience and good knowledge of the local language were hired to conduct interviews. At all sites, research assistants underwent intensive training regarding data collection methods, study procedures, administering the questionnaire (to ensure they understood every question and response category), and maintaining confidentiality of participant data. Research assistants collected data electronically using Open Data Kit (ODK) software. Each interview lasted an average of approximately 20 to 40 minutes. Research assistants often had staggered shifts to include evenings and weekends to allow for calling households when participants were available. Data collectors made three attempts to reach each household before marking the household as unable to be reached; each contact attempt (including unsuccessful attempts and reasons the attempt was unsuccessful) was recorded in call logs. Research assistants often had to set appointments to interview participants, and this was also recorded in the call logs.

Identifiable information was confidentially maintained in secure databases that were accessible only to key personnel from each site. On-site data managers monitored incoming data regularly during data collection to ensure quality and performed minor site-specific data cleaning based on regular debriefings with teams. Data from all sites were pooled and cleaned at the central level using Stata SE 16 to produce multi-site datasets for analysis. All central cleaning was recorded for replicability.

## DISCUSSION

### Challenges encountered.

We faced several challenges with the implementation of our mobile telephone survey among adults, adolescents, and healthcare providers across different SSA study settings in the midst of the COVID-19 pandemic. The adult household survey intended to reach a higher proportion of female respondents because of the survey’s inclusion of nutrition and child feeding questions and evidence that women are often better equipped to answer such questions in SSA.^49^ However, many respondents for the adult household survey were male (63%) because most of the household sampling frames contained the contact information for the head of household, who is typically male in SSA. Upon reaching the male head of household, it was sometimes difficult to request to speak with a female household member, especially if the surveyor was male. In some cases, it was described as inappropriate for a woman to speak on the telephone privately without her husband’s knowledge of the telephone conversation. These difficulties highlight the challenges reaching women through mobile telephone surveys because of cultural and sex norms. To address these challenges and promote high-quality responses for food security and child health modules, we used female interviewers when possible and told interviewers to request to speak with the female household member or to ask the respondent to consult another household member regarding these questions. Overall, the proportion of women (37%) in our sample was comparable to or higher than that of other telephone surveys conducted in similar contexts.^50,51^

Reaching adolescents by telephone was also challenging. Adolescents often were not at home or near the adult household participant when called, which required making appointments to reach them, sometimes using a different telephone number. It was common for adolescents at some sites to request multiple callbacks and sometimes refuse to complete the survey when the scheduled calls were made. To address this, we pursued multiple callbacks at varying times of day; when adolescents were unable to be reached, we continued calling additional households to reach the target adolescent sample size. Our specific focus on adolescents during the COVID-19 pandemic is unprecedented and will provide much-needed data regarding this often-overlooked population group during this crisis.

Privacy and confidentiality issues are a potential concern during mobile telephone studies^52^ and may influence responses, particularly for women and adolescents. To overcome this challenge, respondents were informed of the nature of the survey and the steps taken to ensure confidentiality in the consent script. The survey mode may also mitigate privacy risks by relying almost exclusively on close-ended questions that could be answered in a yes-or-no fashion. In fact, a study of the ethics of mobile telephone surveys in low-income and middle-income countries found that respondents perceive lesser privacy-related risks in mobile telephone surveys compared with face-to-face surveys with the interviewer present.^52^

As in many telephone-based surveys, nonresponse could be an issue. Telephone survey response rates often vary widely across countries and contexts and depend on mobile telephone coverage and whether there is an existing relationship with the participant. The main challenge with response rates is usually reaching valid telephone numbers and getting participants to answer the initial telephone call. Refusals often comprise a low portion of attrition.^53^ In Burkina Faso and Nigeria, we experienced high proportions of nonfunctioning telephone numbers and unanswered calls during the household survey. Approximately 1,200 to 2,600 households were called per site to obtain the target sample size of 300 adults and 300 adolescents. Similar surveys in SSA have also found low response rates. During the Ebola crisis, a survey in Liberia had a response rate of 46% for households that provided a telephone number over the course of five rounds of data collection (26% of the overall sampling frame).^53^ Other CATI surveys had a 50% completion rate in Burkina Faso.^54^ For the healthcare provider survey, challenges obtaining responses were mainly caused by unanswered calls and refusal because of lack of time (especially among physicians). To increase response rates for both the household and healthcare provider surveys, we pursued multiple callbacks at varying times of day.

An additional challenge we encountered involved delays in obtaining ethical approval. In crises, rapid ethical review is required to generate real-time data to enable quick policy responses. However, in many areas, the ethical review processes take months, thus hindering rapid survey efforts, especially for multi-country efforts that require multiple reviews. After the 2014 Ebola outbreak, the World Health Organization Research Ethics Review Committee recommended the constitution of a Joint Ethics Review Committee including representatives from various countries and institutions to expedite research for future outbreaks.^56^ A joint committee would have significantly expedited our survey’s timeline by eliminating the need for multiple reviews and could have also provided a range of expertise and perspectives to strengthen the review process.

### Limitations.

This study had three main limitations. First, study results may not be generalizable beyond the individual communities included in the study because the study sites were not selected to be representative of the larger regional or national populations, thus limiting comparability with data among these populations before the pandemic. Although the findings may not be fully generalizable, we intentionally selected six diverse urban and rural sites to understand COVID-19 impacts and KAP among different populations and communities across SSA.

Although the study design and methods were standardized across sites as much as possible, there were some variations between sites because of the situations in each country and available sampling frames, which may complicate cross-site comparisons. For example, household sampling frames were derived from different sources across sites; therefore, they varied in the amount and accuracy of the information available, with some including a high proportion of dysfunctional or outdated telephone numbers (such as in Nouna, Burkina Faso). In Ouagadogou, Burkina Faso, lists indicated whether households had an adolescent; therefore, we only surveyed adults when an adolescent was also verified as present and able to be interviewed. However, other sites listed households even without adolescents and later called additional households to recruit more adolescents after the adult survey was completed. In addition, households in Ethiopia were approached and only called if consent was obtained in person; however, in non-Ethiopian sites, more respondents than ultimately consented had to be called. The ability to recruit and seek consent in person likely increased survey uptake in the Ethiopian household survey sites. In addition, sites were categorized as urban or rural based on how they are categorized administratively according to the governments of each country and in the existing surveys that were used for each site, which needs to be considered when interpreting urban and rural comparisons across countries.

Another limitation of this survey (and of mobile telephone surveys in general) was potential non-response that might affect the representativeness of the sample to each study community. Respondent selection was limited only to those with access to working mobile telephones, whose contact information was available, who answered the calls, and who were willing to proceed with the telephone survey. We made substantial efforts to increase response rates, including calling potential participants during weekend and evening hours, making three attempts to reach potential participants, and setting appointment times to reach respondents who were less available to answer their telephone. Nonresponse also affected the representativeness of the healthcare provider survey. In Addis Ababa, some professional associations were unwilling to share lists of healthcare providers because of confidentiality reasons; therefore, participants had to opt into the survey using an online link distributed by the professional associations before being called. Even so, the target sample in Addis Ababa was not achieved using this method; therefore, snowball sampling was later used. Similar to the household lists, contact information was sometimes dysfunctional or outdated, and the composition of healthcare provider sampling frames likely did not include all theoretically eligible healthcare providers working at that site. Across all sites, we relied on medical and nursing associations to obtain recruitment lists; therefore, perspectives from additional frontline cadres such as community health workers are absent.

### Strengths and lessons learned.

This survey represents a substantial contribution to the emerging literature regarding the direct and indirect impacts of COVID-19 on different population groups in SSA. A major strength of this study is the inclusion of diverse samples of the general population in both rural and urban areas across multiple countries. Our disaggregation of data by age and sex was also critical because not all countries are reporting COVID-19 data separately for men and women,^57^ and data among adolescents are particularly scarce, thus constraining the understanding of the impacts of the pandemic on different groups.

An important strength is our use of CATI methods, which generate data that are comparable in quality to data collected through in-person interviews and have the lowest attrition of all telephone survey methods.^58^ Compared with online methods and other telephone-based survey methods, such as interactive voice response and SMS, CATI surveys are live and allow for human interaction to clarify questions, build rapport with respondents, and adjust to respondents’ preferred language.^55,59^ Compared with online surveys in particular, CATI surveys can also accommodate illiterate participants and allow for reaching respondents without Internet or with poor Internet connectivity, which can increase representativeness, especially in the contexts of high mobile telephone penetration such as SSA.^55^

An additional strength was our use of the ARISE Network to assemble a team of multidisciplinary experts from across the world with diverse expertise across the survey domains and local context. Although designing and implementing a survey instrument across different study settings in the midst of a pandemic poses a substantial challenge, the ARISE Network collaboration combined skillsets, perspectives, and expertise from local and international partners, which greatly strengthened the study throughout questionnaire design, study planning, implementation, data analysis, and results interpretation.

Overall, our field experiences shed light on important considerations for conducting telephone-based surveys in SSA in the future, especially in the context of a rapidly progressing pandemic. Previous literature indicates that reliable telephone surveys can be completed when mobile telephone ownership rates approach 80%.^55^ Because of the ubiquity of mobile telephone access in many SSA countries, mobile telephone surveys present a valuable and cost-effective supplement to traditional household surveys, which require considerably more resources and time to conduct.^55^ Particularly in times of crisis, rapid data collection and feedback to policymakers are essential. As our study highlights, efforts should be made to maintain complete and accurate databases of telephone numbers that can allow for representative sampling and allow participants to provide consent before sharing their telephone number (unlike databases maintained by telephone service providers). Household data collection efforts (such as large-scale surveys and HDSS) should be leveraged to collect mobile telephone numbers because using household sampling methodologies can increase survey representativeness.^59^ It is also important to regularly update and maintain these telephone lists. As we found during our study, interviewers had to call far fewer telephone numbers to reach target sample sizes when updated telephone lists were available compared with sites with lists that contained many outdated telephone numbers. In addition, efforts should be made to include telephone numbers for multiple family members, not just the household head. As noted, telephone numbers in sampling frames are often for the male head of household, which creates challenges reaching women and adolescents. Further strategies must be developed to ensure increased representation of the experiences of women and adolescents during telephone surveys. Strategies should also be explored to leverage rapid mobile telephone surveys to collect qualitative data, which could shed further light on participants’ lived experiences during the COVID-19 pandemic.

We also found that informing potential participants to expect a call ahead of time may help increase survey uptake. In Ethiopia, response rates for our household survey were much higher because of the in-person household visits conducted before the telephone calls (919 households in Addis Ababa and 388 households in Kersa were called), suggesting the potential benefits of a sequential mixed-mode design when feasible. Previous mobile telephone panel surveys have also reported higher response rates when preceded by a face-to-face visit.^55^ SMS prenotification was only conducted in the two Nigeria sites, thus limiting our ability to draw conclusions about differences in survey uptake with or without SMS prenotification. However, other studies have found that sending an advance SMS resulted in significant increases in response rates, particularly with behavioral messaging aimed to increase intrinsic motivation to complete the survey, suggesting this as a beneficial practice for future telephone surveys.^60,61^ Healthcare providers, in particular, expressed wariness toward completing telephone-based interviews without authorization or advanced notice from their superiors; therefore, future efforts should investigate and leverage both formal authorization and informal channels to maximize health worker participation. Rescheduling calls at convenient times for respondents was also important for maximizing uptake, and staggering interviewer shifts for night and weekend calls was particularly helpful in this regard.

### Future directions.

As COVID-19 continues to affect the health, well-being, and livelihoods of populations across the globe, understanding its impacts on populations and healthcare providers is crucial to designing effective policy strategies to mitigate adverse effects. We will disseminate results from this baseline survey to policymakers in SSA to help them identify COVID-19 impacts and prioritize areas of intervention. Using the mobile platform we established, we plan to extend this baseline survey across other ARISE Network countries and to conduct longitudinal surveillance to continue to generate data from healthcare workers and the general population regarding the impacts of COVID-19. Because mobile telephones are ubiquitous across Africa, virtual interventions, such as digital educational interventions and conditional cash transfers to encourage behavior change, can be scaled up at a low cost to mitigate the consequences of the pandemic across Africa. Based on findings from the baseline survey, we plan to develop, administer, and evaluate such interventions utilizing virtual platforms. These lessons learned will be invaluable for conducting rapid longitudinal surveillance and delivering interventions to mitigate health and socioeconomic impacts in the context of future disease outbreaks.
